# 

**Published:** 1915-12-04

**Authors:** 


### CONTENTS.

PAGE

**A Lay Journal on Medical Education** ... ... 193

**Royal College of Surgeons: Annual Meeting** 194

**Hospital and Institutional News** ... ... ... 195

Gallant Conduct of a Medical Superintendent—The County of London War Hospital—A Splendid Record—Lord Derby and Red Cross Men—A Hospital Treasurer Dies of His Wounds—A Quaker's Military Funeral—Surgeon-General Sir George Makins, K.C.M.G., C.B.—Hospital Sluggards in High Places—The London Jewish Hospital—The L.C.C. and Massage Establishments—Tuberculosis Work in Staffordshire—Boards of Guardians and the Cry for Economy—A Loss to Guy's Hospital—Official Recognition—St. Thomas's Hospital—A "Threatened Injustice" at Birmingham—Lying-In Homes: Their Inspection and Supervision—The Value of Regimental Chiropody—A Recruiting Problem—After-Care Organisation in Bristol—Why the Dispensary is not Superseded—Military Hospitals on the South Coast—Sheffield Students and Latin—Sunderland Royal Infirmary—The Limitation of Hospitals in Leeds—Indiscipline in Sanatoria—This Week's Drug Market.

**The Population Problem** ... ... ... ... 201

The Importance of Maternal Mortality as a Factor.

**Childbed Mortality** ... ... ... ... ... 202

**City of London National Guard** ... ... ... 203

Ambulance Detachment on Duty every Night at St. Bartholomew's Hospital.

**Hospital Weakness and Hospital Strength** ... 203

When the Voluntary Hospital Triumphs.

**National Insurance Act Developments** ... ... 205

The New Medical Benefit Regulations.

**Round the World** ... ... ... ... ... 206

Fellow-Workers and the Roll of Honour.

**The Real King Edward the VIIth.** ... ... 207

Lord Redesdale's Experiences.

[*See over.*]

### MELDRUMS LIMITED,
### TIMPERLEY, MANCHESTER,
BUILD ALL SIZES OF

### REFUSE DESTRUCTORS

*for*

Hospitals, Sanatoria, Infirmaries, and Institutions.

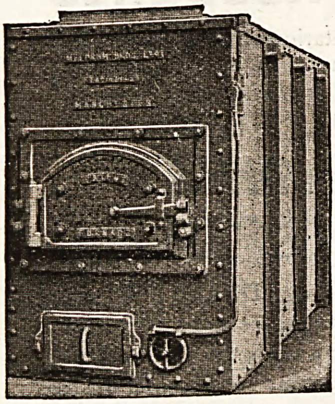

For efficient working and long life there is no plant to equal a Meldrum.

**Also Boiler Furnaces and Stokers for Smoke Prevention and Economy in Fuel.**

*Particulars and Estimates on Application. Enquiries invited.*

### CONTENTS—(*continued*).

| | PAGE |
|---|---|
| **The Institutional Library** ... ... ... ... | 208 |
| Three War Primers—A Surgeon in Khaki—Tropical Hygiene. | |
| **Parliament and the Profession** ... ... ... | 210 |
| First Eastern General Hospital—The Scottish Midwives Bill—Scottish Pharmacists and the Drug Tariff—Wasting the Time of the House—A Case for More Inquiry—Tuberculous Soldiers—Officers of the R.A.M.C. in Egypt—The Irish Apothecaries Again—Unvaccinated Recruits—Who Advises Mr. McKenna?—Army Medical Services Advisory Board. | |
| **The London Hospital...** ... ... ... ... | 211 |
| Memorials to Nurse Cavell. | |
| **Buildings Contemplated** ... ... ... ... | 211 |
| **Patriotism and the Nurse-Training Schools** ... | 212 |
| V. Some Provincial Poor-Law Infirmaries. | |
| **Passing Events and Topics** ... ... ... ... | 213 |
| **Personalia** ... ... ... ... ... ... ... | 214 |
| **Hospital Meetings** ... ... ... ... ... | 214 |
| **Gifts and Bequests** ... ... ... ... ... | 214 |
| **Medical Appointments** ... ... ... ... | 214 |
| **Institutional Needs** ... ... ... ... ... | 214 |
| **The Institutional Worker** ... ... (Suppl.) | 1 |
| Institution Laundries. | |
| Question for December. (Suppl.) | 2 |
| Questions Invited ... .. ... (Suppl.) | 2 |
| Enquiries and Answers. | |
| The Sick and in Need. | |
| Employment and Training. | |
| Miscellaneous. | |
| The Housekeeper's Department. | |

